# Characterization of Mental Illness Among US Coal Miners

**DOI:** 10.1001/jamanetworkopen.2021.11110

**Published:** 2021-05-25

**Authors:** Drew Harris, Timothy McMurry, Amanda Caughron, Jody Willis, Justin C. Blackburn, Chad Brizendine, Margaret Tomann

**Affiliations:** 1 Division of Pulmonary and Critical Care, Department of Medicine, University of Virginia, Charlottesville; 2 Department of Public Health Sciences, University of Virginia, Charlottesville; 3 Black Lung Clinic Program, Stone Mountain Health Services, St Charles, Virginia

## Abstract

This cohort study examines the prevalence of and risk factors for mental illness among current and former coal miners in the United States.

## Introduction

The prevalence of coal mine dust lung disease (CMDLD) in the United States is increasing.^1^ Miners working in central Appalachia are particularly susceptible to rapidly progressive, severe disease.^1^ Treatment options are limited. Management is focused on reducing dust exposure and treating comorbid conditions. Mental illness has yet to be characterized among active and former US coal miners, a population with a high risk of CMDLD.

Anxiety, depression, and posttraumatic stress disorder (PTSD) are known to be underdiagnosed and undertreated in patients with other chronic lung diseases; these mental illnesses have detrimental consequences for functional status, medication adherence, and quality of life.^2^ Studies outside of the United States have found a high risk of mental illness among coal miners.^3^ To our knowledge, this is the first study to describe the prevalence of and risk factors for mental illness among a large population of US coal miners.

## Methods

This cohort study was reviewed by the University of Virginia institutional review board and deemed exempt from a full review and informed consent because data were collected as part of a voluntary survey conducted as part of clinical care. This study followed the Strengthening the Reporting of Observational Studies in Epidemiology (STROBE) reporting guideline. Data were collected between July 1, 2018, and April 28, 2020, and analyzed from May 1 to June 12, 2020.

Stone Mountain Health Services (SMHS) provides medical, behavioral, and legal services for active and former coal miners. In 2018, SMHS implemented mental health screening (MHS) for all patients at the Black Lung Clinic to assess for anxiety (using the 2-item Generalized Anxiety Disorder [GAD-2] screening), depression (using the 9-item Patient Health Questionnaire [PHQ-9]), and posttraumatic stress disorder (using the 4-item Primary Care–PTSD [PC-PTSD 4] screening). Information regarding clinical, physiologic, and radiographic data were abstracted from an existing database, collected for nonresearch purposes.

Continuous data are described using median and interquartile range (IQR); categorical data are described using counts and percentages. Associations between categorical variables were assessed using χ^2^ tests of association. Statistical significance was set at α = .05, and all tests were 2-tailed. All statistical analyses were conducted using R version 3.6.3 (R Project for Statistical Computing).

## Results

During the study period, 2808 of the 2826 US coal miners (99.4%) seen in clinic voluntarily completed a MHS. In the full sample, the median (IQR) age was 66 (60-71) years, 2808 (99.5%) self-identified as White, and 2817 (99.7%) identified as male (Table). A total of 883 of 2364 patients with complete data (37.4%) reported symptoms consistent with major depressive disorder (PHQ-9 score ≥10), including 295 (11.4%) with active suicidal ideation; 1005 (38.9%) had clinically significant anxiety (GAD-2 score ≥3), and 639 (26.2%) had symptoms consistent with PTSD (PC-PTSD 4 score ≥2).

**Table.** Characteristics of Active and Former Coal Miners Who Received a Mental Health Screening Between 2018 and 2020 at Stone Mountain Health Services Black Lung Clinic

Characteristic	Total population, No./total No. (%) (N = 2826)	Definition
Demographic characteristics		
Age, median (IQR), y	66 (60-71)	NA
Race		
White	2808/2826 (99.5)	NA
Other	14/2826 (0.4)	Black/African American individuals; Hispanic/Latino individuals
Sex		
Men	2817/2826 (99.7)	NA
Women	9/2826 (0.3)	NA
Mental illness		
Major depression		
Any	883/2364 (37.4)	PHQ-9 score ≥10
Moderate depression	383/2364 (16.2)	PHQ-9 score 10-14
Moderately severe depression	267/2364 (11.3)	PHQ-9 score 15-19
Severe depression	233/2364 (9.9)	PHQ-9 score 20
Suicidal ideation	295/2599 (11.4)	Yes to question 9 on PHQ-9
Anxiety disorder	1005/2593 (38.9)	GAD-2 score ≥3
PTSD	639/2441 (26.2)	PC-PTSD 4 score ≥2
Pulmonary risk factors and disease		
Smoking		
Current	388/2811 (13.8)	NA
Former	1323/2811 (47.1)	NA
Pack years, median (IQR)	17 (7-30)	NA
Mining tenure		
Worked above and below ground	457/2395 (20.2)	NA
Time above or below ground, median (IQR),y	26 (20-34)	NA
Worked underground only	1398/2395 (61.7)	NA
Time underground only, median (IQR), y	25 (19-33)	NA
Worked above ground mines only	412/2395 (18.2)	NA
Time above ground only, median (IQR), y	28 (7-30)	NA
Spirometry		
FEV_1_/FVC ratio <0.7	906/2482 (36.5)	NA
FEV1, median percent predicted (IQR), %	71.4 (56.4-84.8)	NA
FVC, median percent predicted (IQR), %	99.2 (83.7-114.5)	NA
Chest radiograph		
Coal workers’ pneumoconiosis	1023/1294 (79.1)	Chest radiograph B-read report with profusion score of 1/0+
Progressive massive fibrosis	210/1294 (16.2)	Chest radiograph B-read report with an opacity larger than 10 mm
Currently using supplemental oxygen	508/2735 (18.6)	NA
Other self-reported comorbid conditions		
Hypertension	1828/2762 (66.2)	NA
Sleep apnea	772/2626 (29.4)	NA
Diabetes	761/2711 (28.1)	NA

Abbreviations: FEV_1_, predicted percent forced expiratory volume in 1 second; FVC, forced vital capacity; GAD-2, 2-item Generalized Anxiety Disorder screening; IQR, interquartile range; NA, not applicable; PC-PTSD 4, 4-item Primary Care–Posttraumatic Stress Disorder screening; PHQ-9, 9-item Patient Health Questionnaire; PTSD, posttraumatic stress disorder.

Among MHS participants, 1294 (46.1%) had a chest radiograph, and 2482 (88.4%) had lung function testing during the study period. Of those, 1023 (79.1%) had radiographic evidence of coal workers’ pneumoconiosis (CWP) and 210 (16.2%) had progressive massive fibrosis (PMF). The median (IQR) percent predicted forced expiratory volume in 1 second (FEV_1_) and forced vital capacity (FVC) was 71.4% (56.4%-84.8%) and 91.2% (83.7%-114.5%), respectively (based on Global Lung Function Initiative reference standards). Airflow obstruction, defined by FEV_1_/FVC of less than 0.7, was identified in 906 patients (36.5%).

Compared with patients without hypoxemia, there was a significant association between hypoxemia, defined by current use of supplemental oxygen, and reported symptoms consistent with anxiety (772 of 2052 [37.6%] vs 215 of 451 [47.7%]; *P* < .001), depression (668 of 1883 [35.5%] vs 198 of 408 [48.5%]; *P* < .001), and suicidal ideation (217 of 2065 [10.5%] vs 72 of 452 [15.9%]; *P* < .001). There was no significant association between positive MHS and radiographic or lung function abnormalities.

## Discussion

Mental illness affects the health and well-being of coal miners. In this study of active and former US coal miners, the prevalence of depression (37.4%) far exceeded the prevalence of depression among Medicare beneficiaries in central Appalachia (19.2%).^4^ The rate of suicidal ideation (11.4%) far exceeded the past-year prevalence among US adult men living in West Virginia (3.7%) and Virginia (2.9%).^5^ The prevalence of PTSD (26.2%) was more than 3 times higher than the lifetime rate of PTSD in adults living in rural US counties (7.0%).^6^ These rates of mental illness far exceeded those documented in coal mining populations internationally.^3^

In this population with exceptionally high rates of both CWP (1023 of 1294 patients [79.1%]) and PMF (210 [16.2%]), chronic hypoxemia was associated with anxiety, depression, and suicidal ideation. A limitation of this retrospective cohort study is that causal associations cannot be determined.

Increased assessment of and treatment for unmet mental health needs should be considered for all active and former coal miners. Further study is needed to investigate other risk factors for mental illness in this population, including economic security, substance use disorders, and workplace safety.
